# Description of training loads using whole-body exercise during high-intensity interval training

**DOI:** 10.6061/clinics/2018/e516

**Published:** 2018-10-25

**Authors:** Alexandre F Machado, Alexandre L Evangelista, João Marcelo Q Miranda, Cauê V La Scala Teixeira, Roberta Luksevicius Rica, Charles R Lopes, Aylton Figueira-Júnior, Julien S Baker, Danilo S Bocalini

**Affiliations:** I Laboratorio de Fisiologia Translacional, Departamento de Educacao Fisica, Universidade Sao Judas (USJT), Sao Paulo, SP, BR; II Departamento de Educacao, Universidade Nove de Julho (UNINOVE), Sao Paulo, SP, BR; III Departamento de Biociencias, Universidade Federal de Sao Paulo, Santos, SP, BR; IV Faculdade de Educacao Fisica, Faculdade Praia Grande, Praia Grande, SP, BR; V Grupo de Pesquisa em Performance Humana, Universidade Metodista de Piracicaba (UNIMEP), Piracicaba, SP, BR; VI Institute of Clinical Exercise and Health Science, Applied Physiology Research Laboratory, School of Science and Sport, University of the West of Scotland, Hamilton, Lanarkshire, Scotland; VII Laboratorio de Fisiologia e Bioquimica Experimental, Centro de Educacao Fisica e Deportos, Universidade Federal do Espirito Santo (UFES), Vitoria, ES, BR; VIII Faculdade Adventista de Hortolandia, Hortolandia, SP, Brasil

**Keywords:** Whole-Body Exercise, Training Monitoring, High-Intensity Interval Training

## Abstract

**OBJECTIVES:**

To describe external training load and internal training load through sets of a single session of high-intensity interval training (HIIT) body work.

**METHODS:**

Twenty male individuals (24±3 years) performed a HIIT body work protocol consisting of a single bout of exercise with 1:1 stimuli. The exercises used were 30 min in duration with “all-out” intensity. The exercises included jumping jacks, mountain climbers, burpees and squat jumps, totaling 20 min of exercise. During exercise, total movement capacity, blood lactate measurement, ratings of perceived exertion and recovery, training load and intensity were monitored.

**RESULTS:**

The single bout examined showed a total of 382±89 movements. Differences (*p*<0.01) in the total amount of movement for each exercise were noted, reflecting the difficulty of maintaining exercise over time. Increases in lactate concentrations (before: 0.98±0.16, after: 14.10±1.66; mmol/L) were found postexercise. Significant differences (*p*<0.01) were found after the fifth set, and the values for movement capacity remained higher than the values of the first set, demonstrating high load in a single session. No differences in ratings of perceived exertion (RPE) during the sets were found. However, the ratings of perceived recuperation from the second set were significantly (*p*<0.01) lower than those from the first set.

**CONCLUSIONS:**

The exercise protocol used in this study was of high intensity and produced large values for stress during performance, with increases recorded for the internal load indicators.

## INTRODUCTION

Participation in regular physical activity is associated with many health benefits, which include body fat reduction 1, cardiovascular improvement and lean body mass enhancement 2, the development of increased self-esteem 3 and a more efficient functional capacity 4. For this reason, numerous strategies have been developed to encourage greater participation in physical activity 5. High-intensity interval training (HIIT) is an effective method to reduce insulin resistance 6 and provide improvement in sports performance 7 while promoting positive changes in body composition 8. Although HIIT has been shown to be an effective method to promote physical activity gains, the extent of the benefit is dependent on the quality and quantity of the training stimuli 9.

Recently, the association between HIIT and exercises using whole-body mass or HIIT body work has gained popularity in fitness clubs and among professional athletes. However, knowledge about the adaptations resulting from this exercise modality remains inconclusive 9. The work of Gist et al. 10 demonstrated that HIIT body work led to physiological development and was convenient with regard to time management, cost-effective, and required little operational management compared to traditional training methods. Although this modality is considered simple and easy to use, training load manipulation should be well monitored to guarantee feasible control, efficacy and security 11.

Monitoring of the training load is one of the main factors in the design of physical training programs 12. According to Impellizzeri et al. 13, the training load could be used/implemented in external (ETL) and internal training conditions (ITL). The exact definition of ETL remains unclear and needs clarification, but parameters such as total volume, work completed and time under pressure can be quantified. These values can be obtained using the number of repetitions per overload used, distance covered, number of sprints and amplitudes reached. These values are all frequently used indicators 11. The ITL can be considered an individual-specific physiological adaptation that has occurred as the result of a workload (physical training stress) provided by external ITL. This observation highlights that there are individual physiological and psychological characteristics that respond to external exercise stimuli 13. ITL parameters, such as heart rate variability, oxygen consumption, lactate concentration and subjective perception of effort, are often used to measure responses to exercise 12.

Additionally, the monitoring of training session methods has also been considered an important strategy for adjusting training loads 14,15. The model developed by Foster et al. 16, which quantifies the training load using the subjective perception of effort and the volume of exercise in minutes, has been the most prominent. Nevertheless, there are no experimental studies characterizing HIIT body work using ETL and ITL parameters. Therefore, the objective of this study was to describe ETL and ITL through sets of a single session of HIIT body work.

## MATERIALS AND METHODS

### Sample

After approval by the university research ethics committee (n° 1.738.246/2016), a consent document was signed by twenty healthy adult men (24±3 years) who were physically independent and volunteered to participate in this study. The following parameters were used as exclusion criteria: positive clinical diagnosis of diabetes mellitus, smoking, musculoskeletal complications and/or cardiovascular alterations confirmed by medical evaluation. All of the procedures were in accordance with the ethical standards of the responsible committee on human experimentation (institutional or regional) and with the Helsinki Declaration of 1975, which was revised in 1983.

### Exercise protocols

A single acute bout of high-intensity interval training based on full-body exercise was performed according to Machado et al. 11. Briefly, the training session involved a 5-min warm-up, followed by 20 sets of 30s of all-out exercise and 30s of passive recovery between sets. Jumping jacks, mountain climbs, burpees and squat jumps were used in the protocol.

### Evaluated parameters

#### Anthropometric

Height was measured using a CardioMed (WCS model, Parana) stadiometer, with an accuracy of 115/220 cm. The measurement was performed with the cursor at an angle of 90°, with the patient in a standing position with feet together in contact with the stadiometer. The subjects were instructed to stay in inspiratory apnea, with the head parallel to the ground. Total body mass was measured using a calibrated Filizola electronic scale (Personal Line Model 150), with a 100 g scale and a maximum capacity of 150 kg. Body mass index (BMI, kg/m^2^) was calculated using the equation BMI=weight/height^2^.

#### Total movement capacity

The total amount of exercise movement realized in each set was used as the external training load, as suggested by Machado et al. 11.

#### Blood lactate measurement

Capillary blood samples were collected from a sterile fingertip using a sterile lancet. The first drop of blood was discarded, and freely flowing blood was collected in glass capillary tubes. All blood samples (25 ml) for lactate analysis were evaluated using an Accutrend^®^ (Roche – Basel, Switzerland).

#### Rating of perceived exertion and recovery

Subjects reported their ratings of perceived exertion (RPE) immediately after and before each exercise set, according to Borg 17. Recovery (RPR) was measured with a scale adapted by Laurent et al. 18, with values ranging from 0 to 10. The higher the value is, the greater the perception of recovery by the practitioner.

#### Training load and intensity

Heart rate (HR) was recorded continuously throughout the training session using Polar HR monitors (Polar Oy, Finland). The HR data were recorded every 5s. In an attempt to reduce HR recording error during training, all subjects were asked to check their HR monitors before each session and after each set (∼10 min). Following each training session, the HR information was then downloaded to a mainframe computer using Polar Advantage software.

In addition, an HR-based method of determining workload involved integrating the total volume of the training session with the total intensity of the exercise session. An exercise score for each training bout was calculated by multiplying the accumulated duration in each HR zone by a multiplier allocated to each zone (50% to 60% HRmax=1, 60% to 70% HRmax=2, 70% to 80% HRmax=3, 80% to 90% HRmax=4, and 90% to 100% HRmax=5) and then totaling the results. Maximal heart rate (HRmax) was determined according to the equation by Tanaka et al. 19

The intensity of the single session was measured using the RPE, which was assessed for each subject. The calculation consists of multiplying the duration of the training session in minutes by the exercise intensity, as indicated by the RPE scale 20. Briefly, the subjects were asked to choose a number from 0 to 10 (the maximum value corresponds to the highest physical exertion experienced by the individual, and the minimum value is the resting condition). The subjects were asked to respond to the question: “How was your training today?” at 20 to 30 min postexercise. Additionally, the internal training load was calculated by multiplying the total movements performed in a single session by the RPE.

### Statistical analysis

The D'Agostino–Pearson test was applied for Gaussian distribution analysis. A paired Student's t-test and repeated-measures analysis of variance (ANOVA), followed by Kruskal–Wallis post hoc tests, were performed to compare differences observed during the exercise sessions. An alpha of 0.05 was used to determine statistical significance. All data values were expressed as the means ± standard deviations. All analyses were performed using SPSS software (v 15.0; IBM, Armonk, NY, USA).

## RESULTS

The participants presented no injuries as a result of the workout during or after the exercise session. The biometric parameters assessed are described in Table 1.

**Figure 1:**
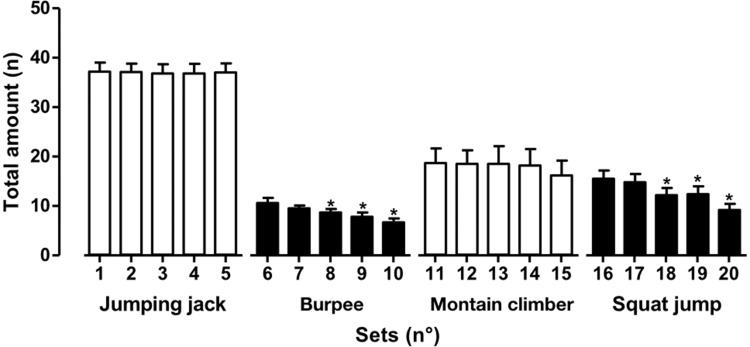
Values are presented as the means ± standard deviations. *p<0.01 *vs.* the first set of each exercise.

Figure 1 shows the total number of movements in each exercise. No differences were found between the sets of exercises. However, differences (*p*<0.01) in the total number (382±89) of movements were found in combined exercises, indicating difficulty in the maintenance of exercise with high metabolic rates.

**Figure 2:**
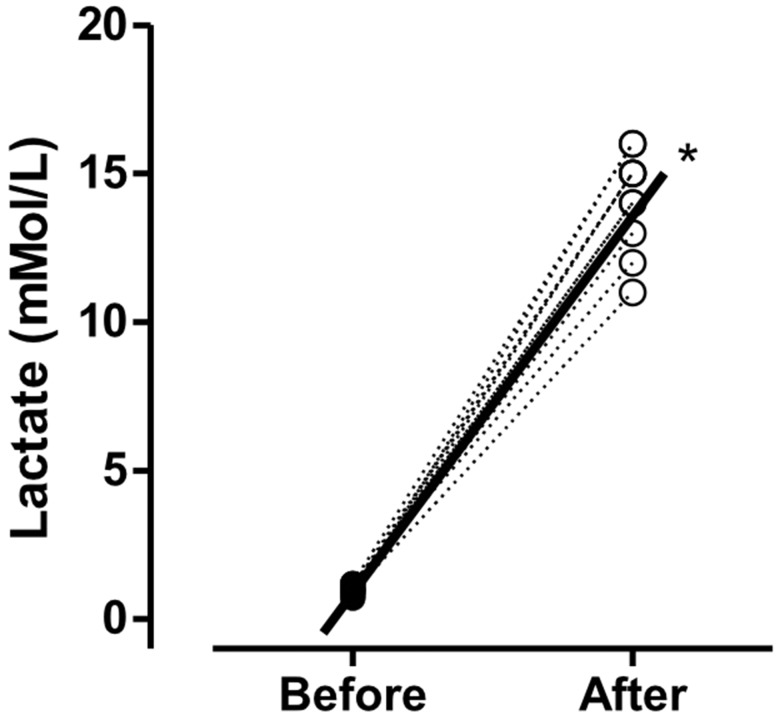
Values are presented as the means ± standard deviations. *p<0.001 *vs*. before.

As shown in Figure 2, significant differences (*p*<0.001) in lactate concentration were found (before: 0.98±0.16, after: 14.10±1.66; mmol/L).

**Figure 3:**
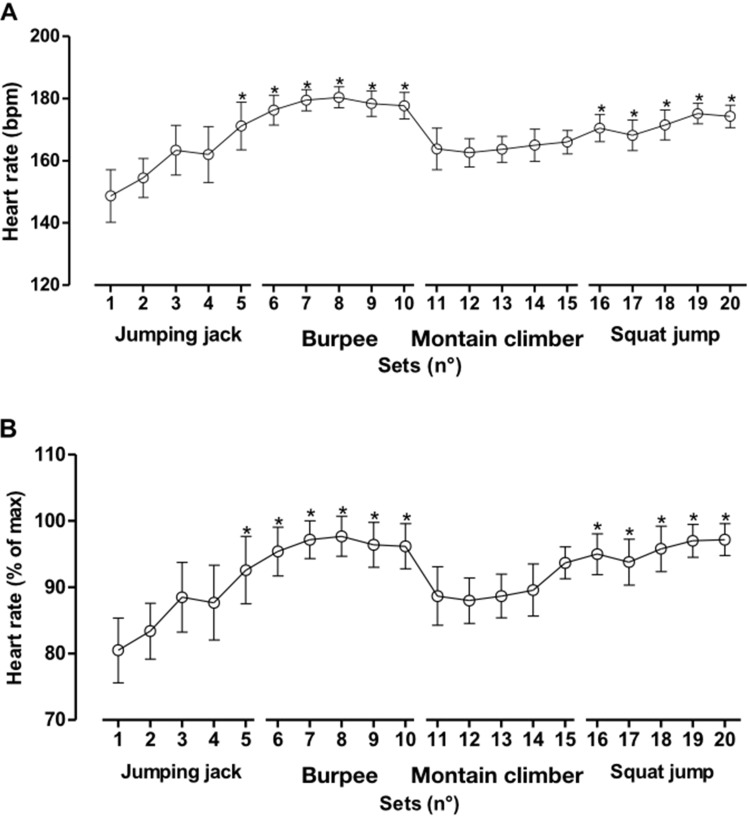
Values are presented as the means ± standard deviations. Panel A: absolute heart rate. Panel B: relative heart rate (% of max). *p<0.001 *vs*. the first set.

As shown in Figure 3, the values for HR (Panel A) and % based on HRmax (Panel B) at 5 to 10 min and at 16 to 20 min were significantly higher than in the first set. As described in Figure 1, these data suggest that combined exercises impair maintenance and require greater efforts due to high metabolic demand. Furthermore, it is possible to state that the first set of the total exercise session, comprised of pattern exercises, did not promote significant changes when compared to combined exercises.

**Figure 4:**
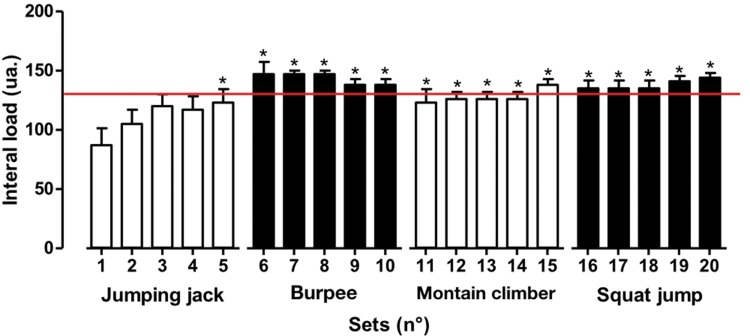
Values are presented as the means ± standard deviations of the rate of internal load immediately after and before each exercise set. *p<0.001 *vs*. the first set.

The internal load of the single exercise session is described in Figure 4. Significant differences (*p*<0.01) were found after the fifth set, and the values remained higher than those of the first set, demonstrating high load in a single session. Additionally, all subjects engaged in high-intensity exercise according to RPE (9.5±0.85), and the internal load characterized by movements performed in a single session and RPE was 3,824±893.

**Figure 5:**
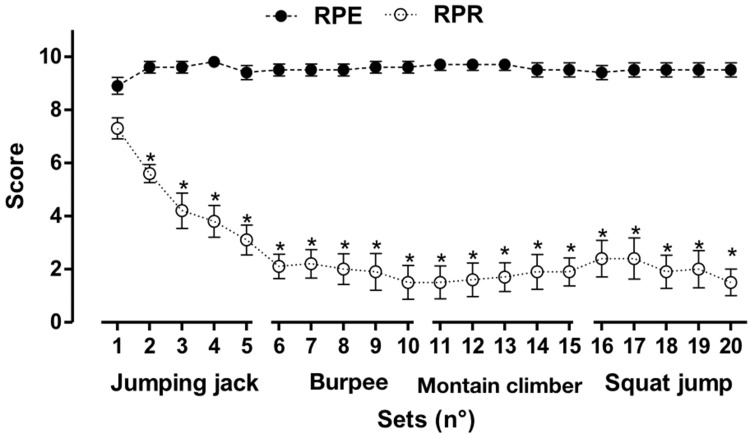
Values are presented as the means ± standard deviations of the ratings of perceived exertion (RPE) and recovery (RPR) immediately after and before each exercise set. *p<0.001 *vs*. the first set.

RPE and the rate of recovery are shown in Figure 5. No differences (*p*>0.05) were found in RPE during the sets. However, the rates of perceived recuperation values from the second set were significantly (*p*<0.01) lower than those from the first set.

## DISCUSSION

The control of loads during the performance of HIIT is challenging for coaches and practitioners when body weight is used. This issue makes it extremely difficult to objectively quantify the external loads used.

Many authors 9,11 have suggested that HIIT exercise programs should be conducted in an *all-out* format. Thus, the subjects should be performing the exercises at maximal intensity, with the highest number of movements and the most repetitions in the exercise period. However, research relating to the immediate physiological responses resulting from this type of activity is scarce. The main findings in the present study correspond to the quantification of variables consolidated in the literature as training load indicators. Our results revealed that the number of movements performed in each exercise series diminished in comparison to the first series for both the burpees and the squat jump exercises.

The blood lactate concentration increased significantly at the end of the exercise period. This finding indicates high glycolytic contribution, metabolic acidosis, and potential fatigue. These metabolic conditions did not impair the performance of jumping jacks and mountain climb series, which indicates that exercises requiring greater muscular strength (burpees and squat jumps) are more prone to the effects of acidosis and muscular fatigue.

Indeed, Fink et al. 21 showed that under high metabolic stress conditions, the number of repetitions made in a subsequent series of strength exercises diminishes drastically. Our results contribute to the development of bodywork HIIT programs, for which the objective is to maintain the number of exercise repetitions during the training session. In addition to the reduction observed in the number of repetitions of the exercises with higher physiological demand, both the burpees and squat jumps provided a greater increase in cardiovascular response than the first series and the mean values recorded, which was not observed for the other exercises.

Although the subjective perception of effort remained near the maximum value of “10” in almost all of the series, characterizing the effort as *all out*, the internal load mean values trended higher for the complex exercises. This result confirms that such exercises (those mobilizing a greater quantity of muscular mass, with greater technical difficulty) call for more strength and greater physiological response when compared to exercises demanding less strength. A possible explanation for this fact, in addition to the metabolic demand previously documented in the literature, could be that the higher-intensity exercises result in more occlusion in the capillary muscles, caused by muscular tension. This mechanism can contribute to an increase in peripheral vascular resistance and, consequently, an increase in HR 22.

Another tool commonly reported in the literature as a viable strategy to control training load is the use of scales of subjective perception of effort. This tool has been used for years as a reliable way to monitor the intensity of workouts 19. Foster et al. 16 pointed out that these scales are ideal for controlling the internal load in cyclical high-intensity activities, because they isolate the specific demands of different forms of training.

In the present study, RPE was maximum in almost all of the 20 series. Additionally, recovery, which was monitored by RPR, fell appreciably following the beginning of the exercise series. Several factors may contribute to these observations: 1) the movement's complexity; 2) the speed demanded in the stimuli (all out); and 3) the short interval for recovery (30s).

Additionally, the exercises used in this study involved integrated actions, such as jumping and pushing, which demand a greater contribution from muscular volume during their execution. This demand is also related to the complexity of the movements involved. Tasks of greater complexity are more liable to generate central and peripheral fatigue, which directly affect the quality of recovery 23, as observed in this study following the burpee exercise. Moreover, the execution of high-speed movements could also be associated with fatigue due to the increase in intensity during the exercise period 24.

It is well documented in the literature 25,26 that short intervals during the recovery phase also contribute to greater residual fatigue between stimuli. This effect is associated both with PCr and ATP concentrations, which stimulate glycolytic metabolism during this type of activity, thereby increasing blood lactate levels 27.

Finally, there are several determinants of involvement in physical activity programs. As such, there are many methods and systems of training to encourage the general population to keep an active lifestyle. Among these training methods, HIIT has been considered an important, time-efficient strategy for improving physical status and body composition. However, there some barriers that should be considered to general prescription, such as those suggested by Gray et al. 28. The use of whole-body-type strategies to perform physical exercise has been considered an important trend by the American College of Sport Medicine. However, there is a lack of information about the physiological impact of this program in the literature. Our study shows for the first time that a single bout of HIIT whole-body training performed at all-out intensity generated considerable acidosis, with different external loads according to physical exercise type (simple and complex characteristics). As such, choosing a physical exercise program should take into consideration each individual motor experience and exercise in order to optimize the best results, considering that the external load is an important parameter for exercise programs. Another interesting point was that throughout the session, the cumulative impact of exercise stimuli directly affected the potential recovery of the subjects during rest. Therefore, parameters of both choice and exercise order may promote different loads (internal and external) during exercise sessions.

## AUTHOR CONTRIBUTIONS

Machado AF, Evangelista AL and Bocalini DS conceived, designed and conducted the study. Miranda JM, Figueira-Júnior A and Bocalini DS also analyzed the data. Miranda JM, Teixeira CV, Rica LR, Lopes CR and Figueira-Júnior A provided critical advice on manuscript writing. Baker JS was responsible for approving the final version of the manuscript for submission.

## Figures and Tables

**Table 1:** Anthropometric characteristics.

Parameters	Mean ± SD	95% of IC
Body mass (kg)	74.0±17.5	61.5-86.5
Height (cm)	1.7±0.1	1.6-1.7
BMI (kg/cm2)	26.3±4.6	23.0-29.6

Values are presented as mean ± standard deviation.
